# State of the psychometric methods: comments on the ISOQOL SIG psychometric papers

**DOI:** 10.1186/s41687-019-0134-1

**Published:** 2019-07-30

**Authors:** Jakob B. Bjorner

**Affiliations:** 1 Optum Patient Insights, Johnston, USA; 2 Department of Public Health, University of Copenhagen, Copenhagen, Denmark; 3 National Research Centre for the Working Environment, Copenhagen, Denmark

**Keywords:** Classical test theory, Item response theory, Rasch measurement theory, Patient-reported outcomes, Depression

## Abstract

**Background:**

Psychometric analyses of patient reported outcomes typically use either classical test theory (CTT), item response theory (IRT), or Rasch measurement theory (RTM). The three papers from the ISOQOL Psychometrics SIG examined the same data set using the tree different approaches. By comparing the results from these papers, the current paper aims to examine the extent to which conclusions about the validity and reliability of a PRO tool depends on the selected psychometric approach.

**Main text:**

Regarding the *basic statistical model*, IRT and RTM are relatively similar but differ notably from CTT. However, modern applications of CTT diminish these differences. In analyses of *item discrimination*, CTT and IRT gave very similar results, while RTM requires equal discrimination and therefore suggested exclusion of items deviating too much from this requirement. Thus, fewer items fitted the Rasch model. In analyses of *item thresholds (difficulty)*, IRT and RMT provided fairly similar results. Item thresholds are typically not evaluated in CTT. Analyses of *local dependence* showed only moderate agreement between methods, partly due to different thresholds for important local dependence. Analyses of *differential item function* (DIF) showed good agreement between IRT and RMT. Agreement might be further improved by adjusting the thresholds for important DIF. Analyses of *measurement precision across the score range* showed high agreement between IRT and RMT methods. CTT assumes constant measurement precision throughout the score range and thus gave different results. *Category orderings* were examined in RMT analyses by checking for reversed thresholds. However, this approach is controversial within the RMT society. The same issue can be examined by the nominal categories IRT model.

**Conclusions:**

While there are well-known differences between CTT, IRT and RMT, the comparison between three actual analyses revealed a great deal of agreement between the results from the methods. If the undogmatic attitude of the three current papers is maintained, the field will be well served.

## Background

Development of a patient-reported outcome measure is a long a complex process involving multiple steps: establishing the domains to be measured and the content of these domains, developing and testing self-report items, determining how items should be combined to form scales, testing scale validity and measurement precision, and developing guidelines for interpretation. The three papers from the ISOQOL Psychometrics SIG focus on only parts of this process, but important ones: The combination of items into scales, and evaluation of measurement properties of the scales based on cross-sectional data. As nicely illustrated by the three papers, different approaches may be taken to evaluate these measurement problems.

**Fig. 1:**
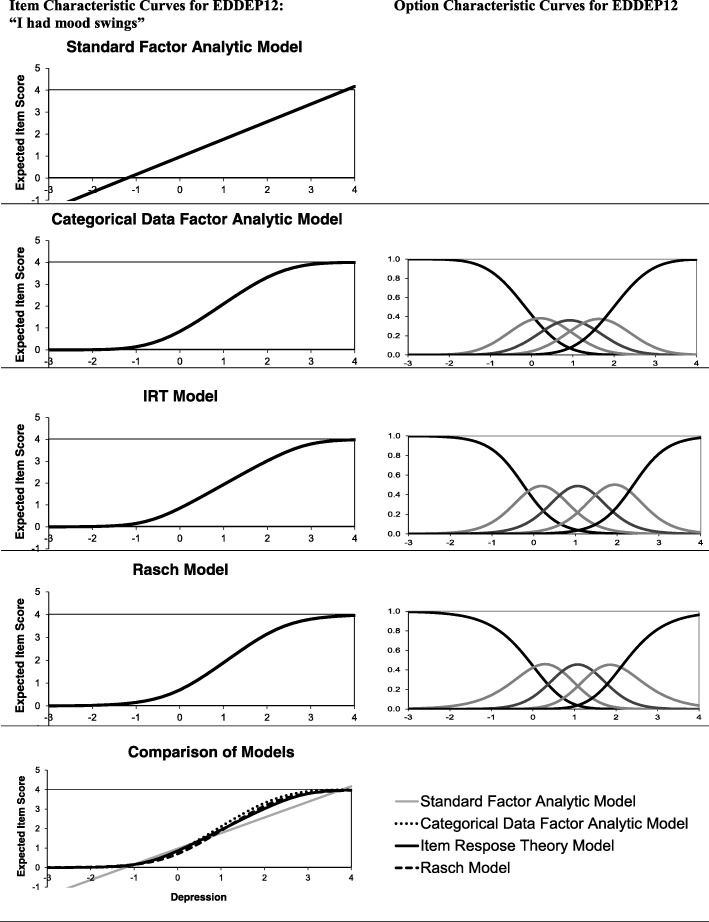
Item characteristic curves and option characteristic curves for EDDEP12 “I had mood swings” according to standard and categorical data factor analytic models, item response theory models, and Rasch models

The papers are very useful in illustrating the reasoning and practical application of three psychometric approaches: classical test theory (CTT) [1], item response theory (IRT) [2], and Rasch measurement theory (RMT) [3]. Since the authors have done all the heavy lifting of describing their approach and applying it to a concrete data set, I can take a global perspective and discuss the similarities and differences between the three approaches. Such differences can have at least three sources: 1) differences in the statistical model used, 2) differences in measurement philosophy, and 3) different habits within each research tradition. The first two types of differences are often (but not always) fundamental, the third less so. Sometimes, different subfields develop approaches that may easily be implemented in other fields but are not traditionally implemented. For example, item and test information functions are usually calculated in an IRT analyses ([2] Figs. 4 and 5), while RTM analysis usually rely on person-item threshold maps ([3] Fig. 1). However, both approaches can be used within both IRT and RMT analyses.

## Main text

### Statistical models

Figure 1 aims to illustrate the different statistical models used in the three papers. The example uses the item EDDEP12 “I had mood swings” with the five response categories “never” (coded 0), “rarely” (1), “sometimes” (2), “often” (3), and “always” (4).^1^ The left column shows the expected (average) item score for different levels of depression. In this example, the depression score is *latent* in the sense that it represents the score we could expect to achieve if we were able to measure depression without error on a continuous interval-level scale from -∞ to +∞ . Zero is set as the mean depression score in the population studied and the unit of measurement is one standard deviation in this population. If depression is normally distributed, 95% of the sample can be expected to be in the interval from − 2 to + 2. This way of setting the metric is typical and was used in the factor and IRT analyses [1, 2]. For ease of comparison, the Rasch model parameters [3] have been re-estimated in this metric.

The top left pane in Fig. 1 shows the implicit model in standard exploratory and confirmatory factor analysis using the product-moments (Pearson) correlation coefficient. This model basically assumes a simple linear regression model: the expected item score is a straight line and the error term around the line has a normal distribution. Figure 1 illustrates the obvious problem when using this model with categorical items: for depression scores below − 1.2 the expected item score is negative and for depression scores above 3.7 the expected item score is above 4. However, since actual item scores only take the values 0,1,2,3,4, the model provides unrealistic predictions. This problem is worst for very skewed items, where a standard linear model provides a very poor fit to the data. While this approach is, by far, the most common in the development of patient reported outcomes measures, Nolte et al. [1] actually avoided it and used a modified approach based on polychoric correlation coefficients [4, 5]. Polychoric correlations are used with ordinal categorical items to estimate what the correlation would have been if the items had been measured on a continuous scale (and assuming a normal distribution for each item on this continuous scale). Categorical data factor analysis using polychoric correlations is equivalent to fitting a so-called normal-ogive IRT model [6]. In this model the probability of answering in category *c* or higher is fitted using the cumulative normal distribution: $P\left({x}_{ij}\ge c\right)={\int}_{-\infty}^{\alpha_i\left({\theta}_j-{\beta}_{ci}\right)}\phi (t) dt$, where *x*_*ij*_ is the response of person *j* on item *i*, *θ*_*j*_ is the latent depression score for person *j*, *α*_*i*_ is a discrimination parameter for item *i*, and *β*_*ci*_ is a threshold parameter for category *c* of item *i.* The discrimination parameter *α*_*i*_ can be calculated from the standardized loading in the confirmatory factor analyses (CFA) model using ${\alpha}_i=\frac{\rho_i}{\sqrt{1-{\rho_i}^2}}$, where *ρ*_*i*_ is the standardized loading for item *i* reported by Nolte et al. [1].

In the second row of Fig. 1, the right pane shows the probability of choosing each item response choice on EDDEP12 for different levels of depression according to the categorical data CFA model. For example, a respondent with a score of zero (the mean of this sample) will have 42% probability of choosing “Never”, 37% probability of choosing “rarely”, 17% probability of “sometimes”, 4% probability of “often” and a negligible probability of choosing “always”. The expected item score (Fig. 1, second row, left pane) can be calculated as the sum of each response choice code weighted by its probability (i.e., for a depression score of zero the expected item score is 0*0.42 + 1*0.37 + 2*0.17 + 3*0.04 + 4*0.003 = 0.84). Figure 1 illustrates that the expected item score for EDDEP12 is always within the range 0–4, making the model more realistic for categorical data than the standard linear model.

The IRT and Rasch models use other approaches to modeling the probability of each response choice. The IRT model used by Stover et al. [2], the *graded response model*, estimates the probability of answering in category *c* or higher using a logistic function: $\mathit{\ln}\left(\frac{P\left({x}_{ij}\ge c\right)}{P\left({x}_{ij}<c\right)}\right)={a}_i\left({\theta}_j-{b}_{ci}\right)$, where *x*_*ij*_ is the response of person *j* on item *i*, *θ*_*j*_ is the latent depression score for person *j*, *a*_*i*_ is a discrimination parameter for item *i*, and *b*_*ci*_ is a threshold parameter for category *c* of item *i.* The graded response model is similar to the categorical factor analytic model in that the basic building block is the probability of answering in category *c* or above. The graded response model uses a logistic link instead of a cumulative normal distribution, but the two models usually given very similar results with *a*_*i*_ = 1.7*α*_*i*_. An expected item score (the item characteristic curve) can be calculated from the option characteristic curves using an approach similar to the calculation for the normal-ogive model.

The Rasch model used by Cleanthous et al. [3] compares the probability of choosing category *c* to the probability of choosing the category below: $\mathit{\ln}\left(\frac{P\left({x}_{ij}=c\right)}{P\left({x}_{ij}=c-1\right)}\right)=\left({\theta}_j-{b}_{ci}\right)$. Also, the Rasch model does not use a discrimination parameter. To facilitation comparison with the other models, the Rasch model used in Fig. 1 estimated a common discrimination parameter for all items $\mathit{\ln}\left(\frac{P\left({x}_{ij}=c\right)}{P\left({x}_{ij}=c-1\right)}\right)=a\left({\theta}_j-{b}_{ci}\right)$ but this does not change the properties of the Rasch model. Finally, Rasch model analysis often estimates an item location parameter, which is the mean of the threshold parameters for the item. Thus, the model can be rewritten $\mathit{\ln}\left(\frac{P\left({x}_{ij}=c\right)}{P\left({x}_{ij}=c-1\right)}\right)=a\left({\theta}_j-\left({l}_i+{d}_{ci}\right)\right)$, where *l*_*i*_ is the location parameter for item *i* and *d*_*ci*_ is the difference between the threshold parameter for category *c* and the location parameter.

The last row of Fig. 1 shows a comparison of the item characteristic curves for the 4 models discussed. The item characteristic curves are rather similar for the categorical factor model, the IRT model, and the Rasch model. These three item characteristic curves differ somewhat from the straight line assumed in standard factor analysis. Thus, while the models are formulated differently, they share some basic properties and provide rather similar expected item scores for item EDDEP12.

The concept of a latent variable (the horizontal axis in Fig. 1) is central in factor analytic, IRT, and Rasch models. However, CTT is built around another concept, *the true score*. The true score can be defined as the expected (i.e. average) score for a particular individual on a particular scale, where the average is assumed to be taken over many independent administrations of the test. The central equation in CTT is *X*_*jk*_ = *T*_*j*_ + *E*_*jk*_ , where *X*_*j*_ is the scale score we observe for person *j* at testing time *k, T*_*j*_ is the *true score* for person *j*, and *E*_*j*_ is a random error for person *j* at testing time *k*. Like the latent variable, the *true score* cannot be directly observed. However, the *true score* differs from a latent variable in several ways: 1) The true score is on the same metric, has the same range, and has the same potential floor and ceiling effects as the observed scale, 2) A true score can be defined for scales where a latent variable is not assumed, e.g. for a composite scale score in a multidimensional instrument. While true scores differ from latent variables in several aspects they are closely associated for unidimensional scales without floor and ceiling effects [7]. Therefore, many psychometric analyses combine methods from classical test theory (e.g. item-total correlations and reliability estimation) with latent variable methods such as factor analytic, IRT, or Rasch models. However, caution should be exercised in interpretation. For example, a high internal consistency reliability does not prove that a latent variable exists [8]. Nolte et al. wisely avoids such interpretations [1].

### Similarities and differences in results from the three approaches

#### Item discrimination

**Fig. 2:**
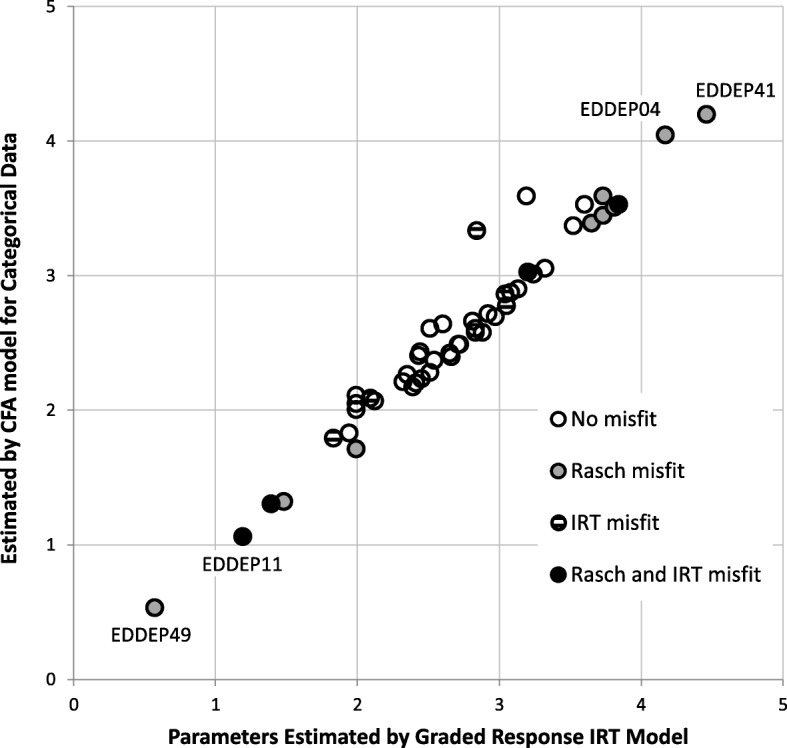
Discrimination parameter estimates according to Confirmatory Factor Analyses (CFA) and Item Response Theory (IRT) models, and item misfit

The comparison of results is based on the analyses of all 51 depression items, which were performed in all three papers. Figure 2 compares the IRT estimates of the discrimination parameters (from [2]) with discrimination parameter estimates calculated from the categorical CFA item loadings (from [1]). The discrimination parameters for the categorical factor analytic model have been calculated from the item loadings using the equation ${a}_i=1.7\frac{\rho_i}{\sqrt{1-{\rho_i}^2}}$ to maximize comparability with the IRT results. Figure 2 shows that the two methods produce very similar parameter estimates. For example, the IRT analysis estimates the discrimination parameter for EDDEP41 “I felt hopeless” as 4.46, while a discrimination parameter estimate of 4.20 was calculated from the CFA model. Similarly, for the item with the lowest discrimination, EDDEP49 “I lost weight without trying”, the discrimination parameter estimates were 0.57 and 0.53, respectively. The correlation between the two sets of parameter estimates is 0.98.

Figure 2 also shows results from the item fit tests available for the IRT and Rasch models. Four items were found misfitting by both models, five items were found misfitting in the IRT model but not in the Rasch model, eight items were found misfitting in the Rasch model but not in the IRT model, while 34 items showed adequate fit to both models. Thus, the two approaches showed only limited agreement on item misfit. Figure 2 illustrates one reason for the lack of agreement: items with very high or very low discrimination parameter estimates in the IRT model were found misfitting in the Rasch model. This is due to the Rasch model’s requirement that all items have equal discrimination. Thus, these differences in item fit are mostly due to differences in the measurement philosophy.

#### Item thresholds/location

**Fig. 3:**
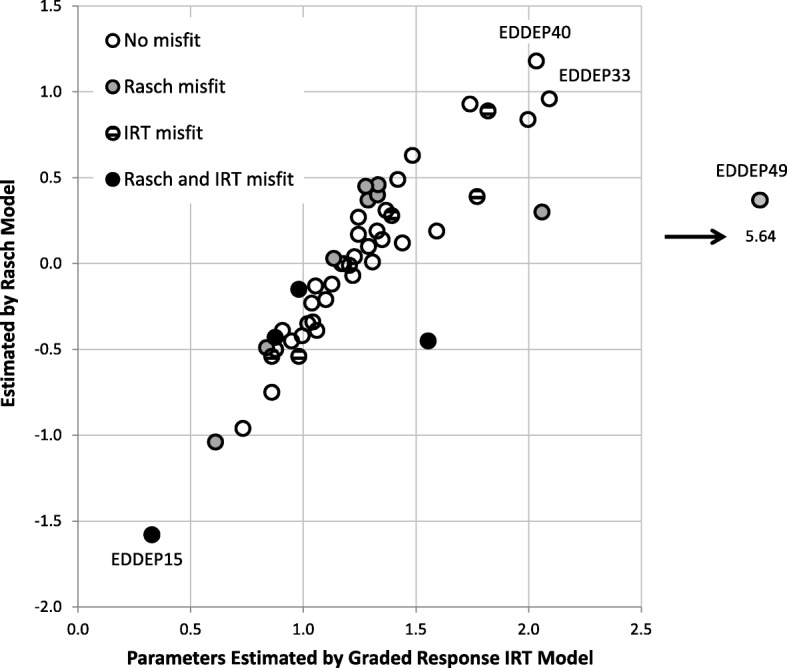
Location parameter estimates according to Item Response Theory (IRT) and Rasch models, and item misfit. For the both models, the item location is calculated as the average of the threshold parameters for that item

Figure 3 compare results from IRT analysis and Rasch analysis regarding item thresholds. For ease of comparison, the IRT results have been summarized as a location parameter, the mean of all the thresholds for that item. The Rasch location parameter estimates are presented using the original scaling of Cleanthous et al. [3] so the metrics are not directly comparable. However, with one exception, Fig. 3 shows high agreement between the location parameter estimates. The correlation between location parameter estimates from the two methods is 0.89. The exception to this agreement is item EDDEP49 “I lost weight without trying” for which the mean IRT threshold was much larger than the Rasch location parameter (Fig. 3). This item also had the lowest discrimination according to the IRT model. The item was flagged as misfitting in Rasch analyses, but not in IRT analyses. The latter lack of significant misfit is probably due to the low discrimination parameter diminishing the statistical power to detect misfit. This illustrates that IRT item fit statistics may not discover all problematic items.

Both approaches identified EDDEP15 “I disliked the way my body looked” as the “easiest” item, meaning that this was the item where respondents indicated most frequent symptoms. Also, this item was flagged as misfitting in both IRT and Rasch analyses. Apart from items EDDEP15 and EDDEP49, no obvious association is found between item misfit and the location parameter.

#### Local dependence

**Fig. 4:**
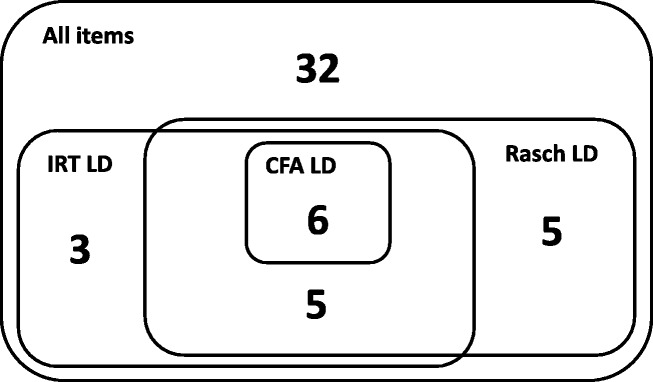
Results from test of local item dependence (LD) using confirmatory factor analysis (CFA), item response theory analyses (IRT) and Rasch analyses

All three approaches evaluated the assumption of local item independence, i.e., that the latent depression variable explains all associations between items. The Venn diagram in Fig. 4 shows a comparison of the results. Using categorical confirmatory factor analysis, Nolte et al. [1] identified local dependence for 6 items. All these items were also identified by IRT and Rasch analyses of local dependence. IRT analysis [2] identified 14 items as locally dependent with other items, 11 of these items were also identified by Rasch analysis [3]. Rasch analysis identified 16 items as locally dependent, of these, five were identified only by the Rasch analyses. However, these five items all had relatively low (below 0.35) residual correlations, while the correlations were higher for all the items where Rasch analysis and IRT analyses agreed. Thus, using a threshold of 0.35 in the Rasch analysis would improve the agreement with IRT analysis considerably. The reported results from categorical CFA and IRT analyses do not permit an evaluation of their criteria for local dependence, but it is highly plausible that the three approaches would agree regarding local item dependence if comparable cut-offs for flagging local dependence were found.

#### Differential item functioning

Both the IRT and the Rasch analysis evaluated differential item functioning (DIF) for gender. The IRT analysis identified 3 cases of uniform DIF (differences in threshold parameters between men and women): EDDEP15 “I disliked the way my body looked”, EDDEP16 “I felt like crying” and EDDEP34 “I had crying spells”. The Rasch analyses identified gender DIF in two items: EDDEP15 and EDDEP16. It is plausible that using other cut-offs for DIF would result in even stronger agreement between the IRT and Rasch analyses. On the other hand, IRT analyses explicitly test for both uniform DIF (group differences in threshold parameters) and non-uniform DIF (group differences in discrimination parameters). It has been suggested that a Rasch analysis may have little power to evaluate non-uniform DIF [9], but the current results do not permit evaluation of this claim. DIF was not evaluated by Nolte et al. [1] and has not traditionally been evaluation inCTT analysis. However, the logistic regression approach to DIF testing [10] is efficient and might be considered a classical approach since it utilizes the sum score rather than an IRT or Rasch model.

#### Measurement precision

**Fig. 5:**
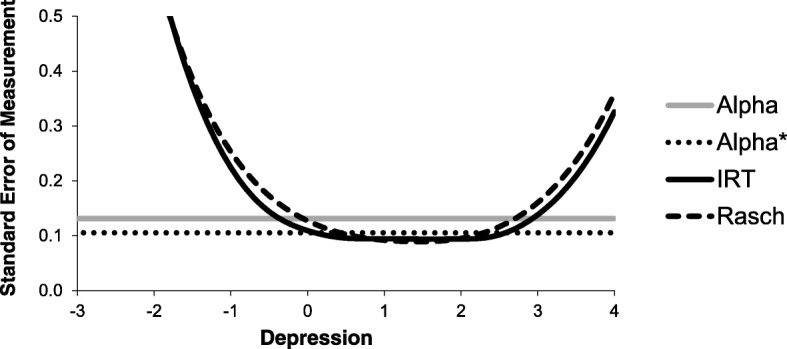
Standard error of measurement for a 51-item depression scale according to Cronbach’s alpha (Alpha), Cronbach’s alpha based on polychoric correlations (Alpha*), item response theory analysis (IRT), and Rasch analysis (Rasch)

All three approaches reported an overall assessment of measurement precision in the form of a reliability coefficient for a scale based on the 51 items. Nolte et al. [1] reported a Cronbach’s alpha coefficient of 0.989 based on polychoric correlations. However, an alpha based on polychoric correlations assesses the reliability we would have achieved if all items were measured on a continuous scale [11]. For this reason, I would prefer reporting the standard alpha coefficient, which in this case is only slightly different, 0.983. Stover et al. report an IRT-based marginal reliability of 0.98 [2], while Cleanthous et al. report a Rasch-based person separation index of 0.95 [3]. However, the IRT and Rasch methods offer a more realistic and detailed way of assessing measurement precision through the analysis of item information functions, test information functions, and standard error of measurement functions. Figure 5 illustrates the differences and similarities between the different approaches to measurement precision, using the standard error of measurement for the 51 items for illustration. To ease comparison, results for the Rasch model have been rescaled to the metric of the IRT model. In CTT, the standard error of measurement can be calculated from the test reliability assuming constant measurement error variance throughout the measurement range. For the depression scale, the standard error of measurement is slightly higher than 0.1. However, IRT and Rasch models do not assume a constant error of measurement. For persons with a high likelihood of scoring either at the floor or the ceiling, measurement error is very high. Figure 5 illustrate a strong agreement between IRT and Rasch models in the assessment of precision throughout the measurement range.

**Fig. 6:**
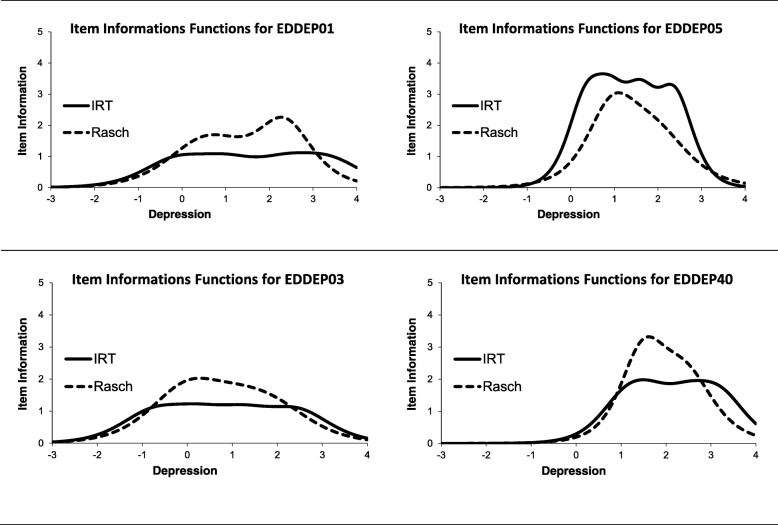
Item information functions for four depression items according to item response theory and Rasch models

The standard error of measurement can be calculated from the test information function which again is the sum of the item information functions. Figure 6 compares the item information functions for 4 items according to IRT and Rasch models. EDDEP01 “I reacted slowly to things that were said or done” has a lower discrimination parameter estimate in the IRT model (a = 1.94) than most of the other depression items. Therefore, over a large part of the measurement range the IRT model assesses item information as lower than the Rasch model. In contrast, EDDEP05 “I felt that I had nothing to look forward to” has a high discrimination parameter estimate in the IRT model (a = 3.52). Therefore, the IRT model assesses item information as higher than the Rasch model does. However, the two approaches agree on the range in which the items are informative. This point is emphasized in the bottom row, which compares an “easy” item, EDDEP03 “I felt that I had no energy”, with a hard item EDDEP40 “I felt that others would be better off if I were dead”. Again, the two approaches agree on the range where an item is informative. The standard error and information functions also illustrate the problem with using the current PROMIS depression items in the general population: the lack of items providing information for the half of the sample with a depression score below 0. If the measure is only used in clinical studies of patients with depression, the lack of measurement precision for scores below 0 is probably irrelevant. However, for some research purposes (e.g. studies of depression prevention) better measurement precision for scores below 0 may be important.

I should also point out that the standard error of measurement functions presented in Fig. 5 might be slightly misleading since they are developed based on 51 items of which several show misfit. Normal practice would be to revise or remove misfitting items, re-fit the model and revise and remove again until acceptable fit, local item independence, and lack of differential item function have been obtained for all items. Only then can item and test information functions be trusted. I deviated from this practice to be able to compare results across the three approaches.

#### Targeting

The issue of scale-to-sample targeting is most frequently discussed within the Rasch framework. To some extent, the analyses of item frequency distributions and floor/ceiling effects in CTT and the analysis of test information function in IRT highlight the same issues. However, person-item threshold maps provide a strong illustration of the reason for floor or ceiling problems. Such maps can also be developed from the results of an IRT analysis or from the results of a categorical data factor analysis (since item thresholds are also calculated in categorical data factor analysis). This is an example of a difference in psychometric practice due to tradition rather than a fundamental difference in measurement philosophy.

#### Category ordering

Response category ordering was evaluated by Cleanthous et al. [3] by examining the order of thresholds. For the set of 51 items, they identified disordered response thresholds in 9 items with the response category “rarely” most consistently not working as intended. Category ordering was not evaluated by the two other analyses, but since this issue may have practical implications, it is worth discussing how category ordering might be evaluated in the two other traditions. In CTT, category ordering is sometime evaluated by asking respondent to place response categories on a visual analogue scale [12]. This approach is simple and not linked to a particular statistical model, but requires separate data collection. In IRT, category ordering can be analyzed using the nominal categories model [13], which does not assume a particular ordering of response categories. The classical specification of the nominal categories model compares the base category (Category 0) to each of the other categories using a logistic link $\mathit{\ln}\left(\frac{P\left({x}_{ij}=c\right)}{P\left({x}_{ij}=0\right)}\right)={a}_{ci}\left({\theta}_j-{b}_{ci}\right)$ where *a*_*ci*_ is the discrimination parameter for each item response category, and *b*_*ci*_ is a threshold parameter for the intersection between the option characteristic curves for the base category and each subsequent category. Thus, the nominal categories model specifies a discrimination parameter for each item response category (except the first). The nominal categories model can be seen as a generalization of the Rasch model for ordinal items. If all differences in discrimination parameters between adjacent item response categories are restricted to equality, the Rasch model for ordinal items is obtained.

**Fig. 7:**
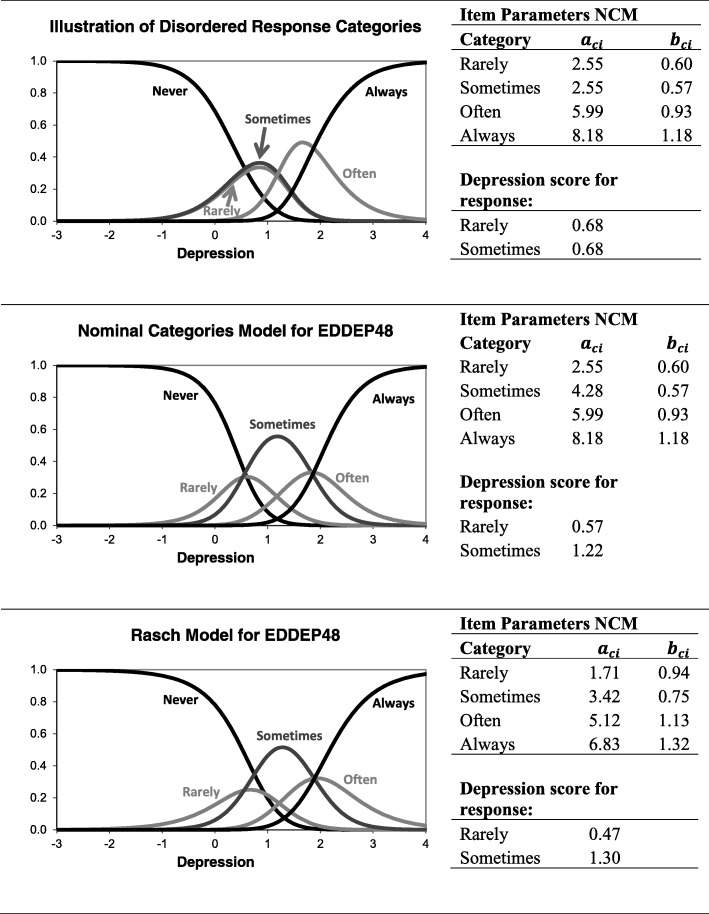
Evaluation of category order for item EDDEP48 using the nominal categories IRT model (NCM)

The top row of Fig. 7 illustrates a case of disordered response categories according to the nominal categories model. The disordered categories can be seen: 1) graphically, where the option characteristic curves for the responses “rarely” and “sometimes” are on top of each other, 2) from the item category discrimination parameter, where both “rarely” and “sometimes” have a discrimination parameter of 2.55, 3) from the depression score estimate, which is 0.68 for both responses (the depression score estimate is calculated as the mean of the area under each option characteristic curve). The second row of Fig. 7 shows the fitted nominal categories model for the item EDDEP48 “I felt my life was empty”, which showed reversed item thresholds in Rasch analysis [3]. However, the IRT analysis supports the conclusion that the response categories are not disordered: the discrimination parameters show an increasing trend and the depression score estimate based on the category “sometimes” (1.22) is clearly higher than the estimate based on the category “rarely” (0.57). The third row of Fig. 7 shows the estimated Rasch model for EDDEP48, using the parameterization of the nominal categories model. The graph shows that for no level of depression the response “rarely” is ever the most likely. This is a clear indication of reversed thresholds according to the Rasch model. However, from an IRT perspective thresholds are irrelevant for conclusions regarding disordered categories; the important issue is the item category discrimination. In this case, the difference in discrimination between each category is constant (and = 1.71), thus strongly supporting the assumed order of categories. From the IRT perspective, an item that fits the Rasch model has no problems with regards to response category ordering, regardless of the ordering of thresholds. This is an example of an issue, where the measurement philosophy differs between the two traditions.

**Fig. 8:**
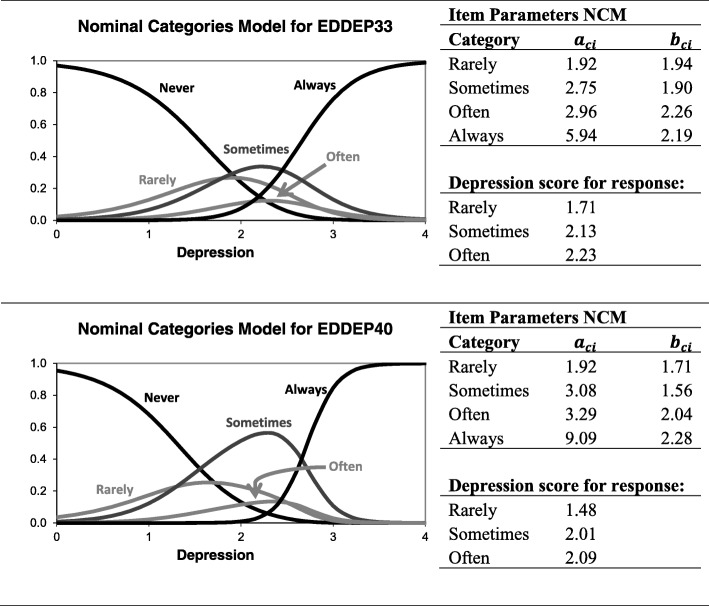
Evaluation of category order for item EDDEP33 and EDDEP40 using the nominal categories IRT model (NCM)

For the 9 items which showed disordered thresholds in the Rasch analysis, a nominal categories model did not show clearly disordered categories for any item. However, two items (EDDEP33 “I thought about suicide” and EDDEP40 “I felt that others would be better off if I were dead”) came close (Fig. 8) and a revision of the response categories may be warranted for these items. For both items the issue seems to be an unclear separation of the categories “sometimes” and “often”. However, it should be mentioned that even though the nominal categories model offers a conceptually clear approach to evaluating the issue of category ordering, the method has shown some problems in practice. Simulation studies have found that the nominal categories model identifies too many cases of disordered threshold in situations where no disorder exists [13, 14]. A slightly revised nominal categories model has shown promise in the assessment of disordered categories [14]. It should also be noted that not all psychometricians in the Rasch tradition agree on the relevance of evaluating threshold reversals (for arguments against the importance of reversed thresholds, see [15, 16], for arguments in support of threshold order analysis see [17]). For all researchers interested in the issue of disordered categories, I strongly recommend the thorough analysis by García-Pérez [13].

## Conclusions

As stated in Donald Patrick’s excellent introduction [18], the authors of the three papers in this series are to be commended for their transparency and rigor in tackling what has been a sometimes contentious debate about different strengths and weaknesses of CTT, IRT, and Rasch analysis. In the spirit of an open discussion of methods, I have aimed to highlight the convergence among methods: all three papers have taken the categorical nature of the items in to account, which is a big improvement from traditional practice. With regards to the identification of local item dependence and differential item function the approaches showed fair agreement and it is likely that even better agreement would be obtained if cut-off points for identification of problems were better matched. The issues of test information functions and scale-to-sample targeting is generally not well treated in CTT, but results from categorical data factor analysis could be used in this regard. Results from IRT analyses and Rasch analyses showed high agreement regarding measurement error and scale-to-sample targeting.

One remaining fundamental difference is the Rasch model’s requirement of equal discrimination for all items. This requirement is based in considerations of test theory (see e.g. [19] for discussion) but can often be motivated on pragmatic grounds: estimates of item discrimination are often noisy and can be influenced by issues like multidimensionality, local item dependence, and general item skewness. Thus, the Rasch model may be chosen for purely pragmatic reasons.

For IRT and Rasch models, the current use of specialized software has both advantages and disadvantages. The specialized packages offer well-established solutions to very demanding estimation tasks but the packages also limit the analytic possibilities. For example, many more tests of fit of the Rasch model are available [20] than are currently implemented in standard Rasch programs such as RUMM [21]. On the other hand, optimal estimation procedures for the Rasch model (conditional maximum likelihood estimation) are not implemented in IRT programs like IRTPRO [22]. Thus, the specialized software packages direct psychometricians to the specific analytic strategy supported by the particular package. For this reason, the development of multiple analytic possibilities within software packages (such as the inclusion of IRT capabilities in the factor analytic package MPlus [23]) and the development of advanced psychometric capabilities in open-source packages such as R are encouraging signs. If psychometricians are provided more flexibility in choosing the analytic strategy that fits the problem, and the undogmatic attitude of the three current papers is maintained, the field will be well served.

## Data Availability

The datasets mentioned in this article are available from the PROMIS Health Organization, http://www.healthmeasures.net/explore-measurement-systems/promis

## Abbreviations

CFA: confirmatory factor analyses
CTT: classical test theory
DIF: differential item functioning
IRT: item response theory
LD: local item dependence
NCM: nominal categories IRT model
RMT: Rasch measurement theory

## Footnotes

1 The three papers use different labeling of the items. Here the labeling from Nolte et al. [1] is used throughout.
